# The Diagnostic Potential of Non-Invasive Tools for Oral Cancer and Precancer: A Systematic Review

**DOI:** 10.3390/diagnostics14182033

**Published:** 2024-09-13

**Authors:** Tania Vanessa Pierfelice, Emira D’Amico, Chiara Cinquini, Giovanna Iezzi, Camillo D’Arcangelo, Simonetta D’Ercole, Morena Petrini

**Affiliations:** 1 Department of Medical, Oral and Biotechnological Sciences, University “G. d’Annunzio” of Chieti-Pescara, Via dei Vestini 31, 66100 Chieti, Italy; tania.pierfelice@unich.it (T.V.P.); emira.damico@unich.it (E.D.); gio.iezzi@unich.it (G.I.); camillo.darcangelo@unich.it (C.D.); simonetta.dercole@unich.it (S.D.); 2 Department of Surgical, Medical, Molecular Pathologies and of the Critical Needs, School of Dentistry, University of Pisa, 56100 Pisa, Italy; chiara.cinquini@gmail.com

**Keywords:** non-invasive diagnostic tools, oral cancer, oral precancer, vital staining, oral brush, light-based technology, spectroscopy

## Abstract

Objectives: This systematic review aimed to analyse the published evidence for the use of non-invasive methods for the early detection of oral squamous cell carcinoma (OSCC) and oral potentially malignant disorders (OPMDs). Methods: The literature was systematically searched through several databases: PubMed, Cochrane Library, and Web of Science. Additional exploration was performed through cross-checks on the bibliographies of selected reviews. The inclusion criteria involved studies assessing the application of non-invasive tests on humans in the screening, diagnosis, or surveillance of OSCC or OPMDs and reporting sensitivity (SE) and specificity (SP). The Newcastle–Ottawa scale (NOS) was applied to assess the quality of the studies included. Results: The search strategy resulted in 8012 preliminary records. After a duplicate check, 116 titles remained. After abstract analysis, 70 papers remained. After full text analysis, only 54 of the 70 papers fit the inclusion criteria (28 were original articles and 26 were reviews). Those 26 reviews were used to manually search for further original articles. From this last search, 33 original articles were found. Thus, a total of 61 original studies were included and investigated. Findings from this systematic review indicate useful information, such as a description of the mechanisms, ease of use, limitations, and SE and SP values, to drive the choice of the optimal minimally invasive method to be utilized as an adjunctive tool to examine the suspicious lesions. Conclusions: Each of the analysed tools can be improved or implemented, considering their high SE and low SP. Despite advancements, incisional biopsy continues to be the gold standard for the definitive diagnosis of oral cancer and precancerous lesions. Further research and development are essential to improving the sensitivity, specificity, and reliability of non-invasive tools for widespread clinical application.

## 1. Introduction

Oral cancer is a specific type of head and neck cancer that originates in the tissues of the mouth and can affect the tongue, gums, lips, inner cheek lining, floor of the mouth, and roof of the mouth (palate) [1]. Oral cancer is a significant public health concern, with an estimated 600,000 new cases and 300,000 deaths worldwide annually [2]. The five-year survival rate for oral cancer remains relatively low, at around 50%, primarily due to late detection [3]. Oral squamous cell carcinoma (OSCC) accounts for a substantial portion of oral cancers. Globally, OSCC is estimated to affect over 377,000 individuals annually, with a higher prevalence in developing countries [2]. Oral potentially malignant disorders (OPMDs) refer to a broad category of distinct histological lesions that have the potential to progress to OSCC [4]. The most common OPMDs include oral leukoplakia (OL), oral erythroplakia (OE), oral lichen planus (OLP), oral submucous fibrosis (OSF), and oral epithelial dysplasia (OED), each of which has diverse risks of malignant transformation [4,5]. OL appears as white plaques, and it can be clinically distinguished as homogeneous or non-homogeneous. The non-homogeneous type (erythroleukoplakia) often has a white/red appearance and presents a higher risk for malignant transformation [4,5]. OE appears as a single erythematous oral mucosal lesion, and among OPMDs it has the highest risk of becoming malignant [6]. Among OPMDs, the OLP is the most prevalent, with a prevalence of 1–2% in the general population [7]. Clinical aspects of OLP can be reticular, plaque-like, bullous, atrophic, or erosive, and the malignant transformation rate is very low (<1–6%) [4]. OSF is a chronic disorder of the oral cavity characterized by collagen deposition and strong fibrosis, with a medium-high potential for malignant transformation (rate: 7–30%) [4]. OED is a spectrum of epithelial abnormal changes linked to an increased risk of transition to cancer, and it is graded based on a histologic grading system: WHO Histologic Grading of Oral Dysplasia [2].

The majority of current methods for predicting potential malignancy are based on clinical data, and they take into account factors including non-homogeneity, anatomical location, age, gender, the presence of dysplasia, and tobacco use [8]. The early detection of oral cancer and precancerous lesions is crucial for improving patient outcomes. A clinical oral examination (COE) is the cornerstone of oral cancer screening and a crucial component of any dental visit, consisting of inspection and palpation to detect oral mucosal changes [9]. In a systematic study, Epstein et al. evaluated the sensitivity and specificity of COEs in identifying OSCC and OED, finding that values were 93% and 31%, respectively [9]. This suggested that there were a lot of false-positive events, probably due to misunderstandings among clinicians. A definitive diagnosis is accomplished by performing a biopsy and histological assessment, which represents the Gold Standard for diagnosing oral cancer and precancer. This process involves a pathologist collecting a small sample of suspicious tissue from the oral cavity and examining it under a microscope [10]. While highly reliable, this method comes with some drawbacks, such as invasiveness, pain, cost, and time consumption. The biopsy procedures require breaking the skin or mucous membrane, which can be uncomfortable and potentially lead to bleeding or infection. Depending on the location and type of biopsy, some degree of pain or discomfort is likely during the procedure. The biopsy approach implies pathologist fees and lab analysis, making these procedures expensive. The entire process, from biopsy to diagnosis, can take several days to weeks, delaying treatment initiation [10]. The development of non-invasive diagnostic tools for oral cancer and precancer has the potential to revolutionize early detection and improve patient outcomes. These tools offer several advantages over traditional methods, including non- or minimal invasiveness, objectivity, ease of use, and cost-effectiveness. Non-invasive tools do not require tissue sampling, reducing patient discomfort and the risk of complications. Non-invasive tools can provide objective data that can be quantified and analysed. Many non-invasive tools are portable and easy to use, allowing for screening in a variety of settings, including dental offices and community health centres. Non-invasive tools can be more cost-effective than traditional methods, particularly for large-scale screening programs [11]. In recent years, there has been significant progress in the development of non-invasive diagnostic tests for oral cancer and precancer. These tests can be broadly categorised into the following groups: Vital Staining, Light-Based Technology, Oral Brush, and Spectroscopy [10]. This systematic review attempts to assess the literature on the efficacy of non-invasive methods in the detection of OSCC and OPMDs in order to support dentists in using these diagnostic techniques during clinical practice, taking into account factors such as sensitivity (SE) and specificity (SP).

## 2. Materials and Methods

**Figure 1:**
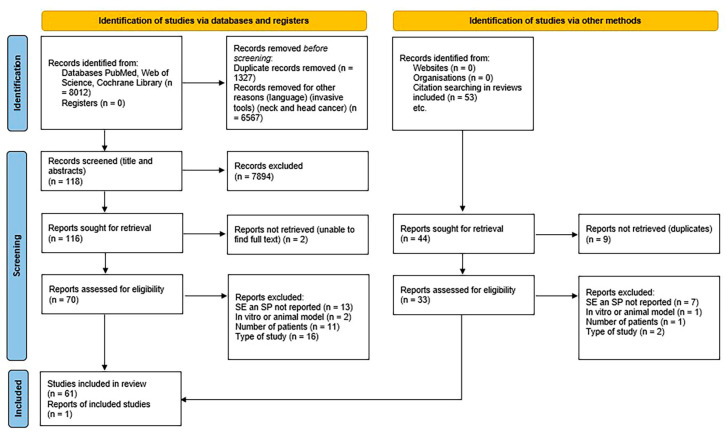
Flow-chart diagram for the selection of the 61 studies included in the present analysis, according to “The PRISMA 2020 statement: an updated guideline for reporting systematic reviews.” The document can be accessed at the following link: https://www.prisma-statement.org/prisma-2020-flow-diagram (accessed on 5 August 2024).

The protocol of this review has been developed according to the PRISMA (Preferred Reporting Items for Systematic Review and Meta-Analyses) statement [12,13] (Figure 1).

### 2.1. Search Strategy

Three independent researchers (TVP, ED, and CC) conducted a search strategy with the following keywords: “oral cancer AND non-invasive diagnostic tools” OR “oral precancer AND non-invasive diagnostic” OR “oral cancer AND non-invasive diagnosis” OR “oral precancer AND non-invasive diagnosis” OR “oral cancer AND brush” OR “oral precancer AND brush” OR “oral cancer AND cytology diagnosis” OR “oral precancer AND cytology diagnosis”. The search was conducted on different electronic databases, including PubMed, Web of Science, and Cochrane Library.

### 2.2. Eligibility Criteria

The inclusion criteria were:

(1) Papers published between 2014 and 2024;
(2) Papers in the English language;
(3) Studies conducted on humans;
(4) Studies with more than 10 patients included;
(5) Papers that reported sensitivity (SE) and specificity (SP) percentages.

The exclusion criteria were:

(1) Studies in vitro, or performed on animal models;
(2) Studies that analyse COE or invasive diagnostic tools (e.g., scalpel biopsy) alone;
(3) Studies that distinguish sensitivity and specificity data based on the different grades of lesions (e.g., mild dysplasia);
(4) Studies that analyse salivary biomarkers;
(5) Case reports, case series with less than 10 patients, conference proceedings, personal communications, editorials, or descriptive studies;
(6) Studies also including tumours of other head and neck regions (e.g., oropharynx).

### 2.3. Selection Process

The presence of duplicates was assessed through Mendeley software 2.109.0. Two independent expert researchers (TVP and ED) screened the titles only; in a second round, they also included abstracts. After a duplicate check, 116 titles were saved. After abstract analysis, 70 papers were saved. In a third round, two independent expert researchers performed a full-text analysis, taking into account the following inclusion and exclusion criteria. After full-text analysis, only 54 of the 70 papers fit the inclusion criteria (28 were original articles and 26 were reviews). The 26 reviews (Table 1) included in the full-text analysis were consulted to perform a further manual search and to find other original manuscripts for the qualitative analysis. From this last search, 33 original articles were found. Thus, a total of 61 original studies were included and investigated.

### 2.4. Data Items

The systematic review was designed by two reviewers (GI and MP) to answer the following focused questions:

- What are the different types of non-invasive diagnostic tools available for oral cancer screening? What are the underlying principles behind each method?
- How do these tools work? Are there any limitations for each tool?
- How easy is each class of tools to use? Do they require specialised training for healthcare professionals?
- What is the individual non-invasive methods’ diagnostic accuracy (sensitivity and specificity)?
- What are the future directions for researching non-invasive diagnostic tools for oral cancer?

### 2.5. Study Risk of Bias Assessment

The quality of the cohort, cross-sectional, and case–control studies eligible for inclusion was evaluated by three reviewers (TVP, ED, and MP) using the Newcastle–Ottawa Scale (NOS) [40]. Studies with NOS scores 0–3, 4–6, and 7–9 were considered of low, moderate, and high quality, respectively. The protocol of the systematic review was registered with the OSF register DOI: doi.org/10.17605/OSF.IO/NVCKQ. The review protocol can be accessed at the following link: https://osf.io/by27q/ (Date registered, 15 July 2024).

### 2.6. Effect Measures

For each study, the typology of the study and diagnostic tools, the number of lesions and patients, the number of pre-cancer and cancer lesions, the values of SE and SP, the positive predictive value (PPV) and negative predictive value (NPV), and the main conclusions were evaluated. In addition, the mean and standard deviation (SD) of SE and SP for each diagnostic tool were calculated, using the reported percentages in each study.

## 3. Results

### 3.1. Selection of the Manuscripts

The electronic search identified 8012 titles: 7059 manuscripts from PubMed, 625 from Web of Science, and 328 from Cochrane Library. After duplicate removal and title screening, 116 titles were selected. Among them, 70 abstracts were retained. Two reviewers (TVP and ED) conducted abstract revision, and any discrepancies were overcome through a discussion between the two auditors, which led to a common solution. From these abstracts, a total of 54 manuscripts met inclusion criteria, and full-text analysis of 28 original articles and 26 reviews was performed. From the 26 reviews listed in Table 1, an additional manual search was conducted to find 33 other original articles. Thus, in total, 61 original manuscripts were included and are listed in Table 2. These 61 papers include 3 randomized clinical trials (RCT), 23 cohort studies, 22 cross-sectional studies, and 13 case–control studies.

### 3.2. Description of Characteristics, Results, and Quality of Each Study

The findings from this review are subdivided according to the original questions. The risk of bias within and across studies is addressed briefly, and the consensus NOS quality scores for each study are displayed in Supplementary S1 (Tables S1–S4). Among the 23 cohort studies, 3 were considered of high quality, 18 of moderate quality, and 2 of low quality (Tables S1 and S4). The 22 included cross-section studies showed moderate quality in 21 studies and low quality in only 1 study (Tables S2 and S4). The case–control studies (*n*= 13) contained 5 of high quality and 8 of moderate quality (Tables S3 and S4).

### 3.3. What Are the Different Types of Non-Invasive Diagnostic Tools Available for Oral Cancer Screening? What Are the Underlying Principles behind Each Method? 

**Figure 2:**
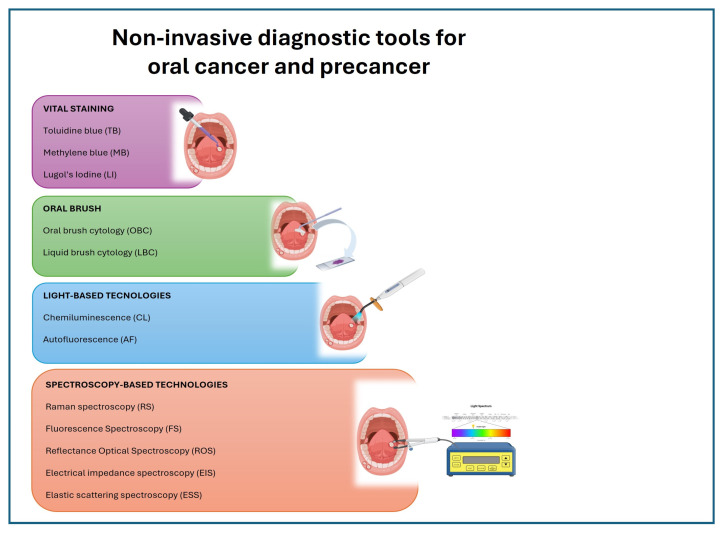
Illustration of non-invasive tools for the diagnosis of oral cancer and precancer, grouped by their class. Created with BioRender.com and Microsoft(R) PowerPointR for Microsoft 365 MSO (Version 2406).

Diagnostic tools are listed in Table 3 and grouped into four classes (Figure 2) based on the underlying principle behind each method.

#### 3.3.1. Vital Staining

Vital staining offers a non-invasive and potentially cost-effective approach to aiding in the detection of oral cancer and precancer. This technique uses dyes that selectively stain living cells with abnormal characteristics. These dyes are applied directly to the oral cavity and interact with specific cellular components in suspicious lesions [22].

#### 3.3.2. Oral Brush (OB)

Brush cytology is a simple, non-invasive method for the early diagnosis of many epithelial cancers, including oral cancer. Brush cytology is particularly useful for diagnosing dysplasia and early carcinoma in patients who are asymptomatic or those with minor symptoms that do not warrant immediate biopsy [39]. The mechanism of cytology is based on the fact that dysplastic and cancer cells tend to have fewer and weaker connections to each other and to nearby normal cells in the surrounding tissue. Dysplastic and cancer cells, therefore, tend to “slough off” or exfoliate preferentially and can be easily collected from the surface of the lesion [39,66].

#### 3.3.3. Light-Based Technology

Light-based techniques, including chemiluminescence (CL) and autofluorescence (AF), work by analysing the differences in light absorption and fluorescence between healthy and abnormal tissues as consequences of abnormal metabolic processes and alterations that influence the absorbance and autofluorescence properties of cancerous and precancerous cells [21].

#### 3.3.4. Spectroscopy-Based Technology

Spectroscopy-based technologies offer a promising alternative, providing a non-invasive approach for the diagnosis of oral cancer and precancerous lesions. Spectroscopy-based technologies are able to detect alterations in tissue composition often associated with precancer or cancer [24]. The complicated process from normal tissue to cancer progression involves the accumulation of molecular and cellular alterations. The morphological and biochemical abnormalities in the malignant tissue, such as the thickening of the epithelium, the expansion of the nucleus, and modifications to the extracellular matrix (ECM) architecture, have become chemical changes detectable by optical spectroscopic methods. By analysing the specific light patterns, optical spectroscopy, which includes Raman spectroscopy (RS), fluorescence spectroscopy (FS), reflectance optical spectroscopy (ROS), and elastic scattering spectroscopy (ESS), can potentially identify these biochemical changes [102,103]. The above techniques expose oral tissue to light at different wavelengths. The way this light interacts with the tissue, including through absorption, scattering, and reflection, results in changes on the basis of alterations in tissue composition often associated with precancer or cancer [24]. Spectroscopy-based technology also includes electrical impedance spectroscopy (EIS), which provides an electrokinetic profile of cells and can distinguish healthy cells from pathological cells based on their dielectric properties (impedance and capacitance). All these techniques have the potential to diagnose early-stage cancer and dysplasia in real time with high sensitivity, without causing patient discomfort, and they are potentially effective in analysing bodily fluids.

### 3.4. How Do These Tools Work? Are There Any Limitations for Each Tool?

Toluidine Blue (TB) is the most commonly used vital stain for oral cancer screening [41,44,45]. Healthy tissues stain minimally, while precancerous and cancerous lesions appear blue due to increased cellular uptake of the dye. TB can be applied as a topical solution or mouth rinse. After rinsing, stained areas are identified and assessed. The intensity and pattern of staining can provide clues about the nature of the lesion [32,49,50]. 

Methylene Blue (MB), similar to toluidine blue, is a dye with acidophilic properties; it penetrates cells with abnormal increases in nucleic acid, resulting in differential absorption between normal cells and highly dysplastic/malignant cells, which exhibit increased metabolic activity and a higher content of nucleic acids. This allows them to bind more of the dye, making it appear blue when stained. Methylene blue is generally considered to have a less toxic profile compared to toluidine blue, which can be a factor for some patients [43]. Despite variability in the reported diagnostic efficacy of MB staining, it is considered an important adjunct to COE, with high sensitivity properties [42,46,47]. 

Lugol’s Iodine (LI), a solution of iodine and potassium iodide (I2-IK), joins the ranks of vital stains used in the detection of oral cancer and precancer. It offers a non-invasive and potentially cost-effective approach to aiding early diagnosis, similar to methylene blue and toluidine blue. Lugol’s iodine exploits a different staining principle, unlike methylene blue and toluidine blue. Healthy oral mucosal cells contain glycogen, a carbohydrate that readily binds to iodine, turning brown upon contact. Precancerous and cancerous cells often exhibit a reduced capacity for glycogen storage [104]. This means they take up less iodine, appearing unstained or light brown compared to healthy tissue. While not as widely used as methylene blue or toluidine blue for general screening, Lugol’s iodine may be used in specific contexts to identify precancerous lesions. Recently, Xia CW et al. reported that I2-IK staining improved the micro-CT image quality to drive the surgical margin for tongue squamous cell carcinoma (TSCC) specimens [105]. Among the tools described in this study, vital staining tests represent the cheapest and easiest methods, but they cannot replace definitive diagnostic methods like biopsy; they can just serve as a cost-effective and patient-friendly screening method.

Oral brush cytology (OBC) is a conventional method for dentists and healthcare professionals in the fight against oral cancer, offering a minimally invasive way to assess suspicious lesions and identify potential concerns. This method utilizes a softer brush designed to collect exfoliated cells that have naturally shed from the surface of the oral cavity. The collected cells are evaluated for abnormalities, which can suggest the presence of cancerous or precancerous lesions. This technique may indicate the presence of abnormal cells but cannot definitively confirm cancer, and it may miss underlying abnormalities present in deeper tissue layers [52,54,56,57,58,64,67,68]. During the procedure, the scraped cells are directly smeared on a glass slide. This method requires proper techniques because mishandling can alter the morphology of the collected cells [39]. Ma JM et al. applied Feulgen staining to exfoliative cells obtained from cytobrush, to measure Nuclear DNA contents (ploidy) with an automated DNA image cytometer. The authors reported that the combination of brush cytology with DNA-image cytometry is a useful method for monitoring potentially malignant oral disorders [52].

Liquid-based brush cytology (LBC) offers significant advantages compared to conventional exfoliative cytology, particularly regarding the good quality of preparation of cell morphology and staining, and a clean background. During the technique process, a thin layer of cells is created on the slide by spreading the cells in a fixative solution, allowing a better quality of preparation, a greater cell collecting capacity, and fewer specimen artifacts caused by blood and saliva [39,106]. However, several days are required to obtain results from LBC [55,60,63]. LBC is also not a reliable means of evaluating lesions with thick keratin layers. 

Chemiluminescence-based devices (ViziLite^®^, ViziLite^®^ Plus, MicroLuxTM/DL) are used after rinsing the mouth with acetic acid. This combination leads to the coagulation of cellular proteins and cell dehydration on the surface, with a consequent reduction in epithelium transparency. Abnormal cells appear white under blue-white illumination, while normal cells appear blue [107]. Currently, the main limit of ViziLite^®^ is the high percentage of false-positive and false-negative results obtained in the identification of dysplastic areas. In any case, ViziLite^®^ facilitates the identification of hyperkeratotic areas and may increase the visibility of mucosal lesions.

Autofluorescence-based devices are used to illuminate fluorophores such as Nicotinamide adenine dinucleotide (NADH), flavin adenine dinucleotide (FAD), cellular coenzymes, collagen, and elastin, which are contained in the cells of connective tissues. In normal tissue, when excited by blue/violet light at a specific wavelength in the range 400–450 nm, these fluorophores emit green light. Loss of autofluorescence has been associated with malignant and pre-malignant lesions. Typically, healthy tissue appears green, while abnormal tissue appears dark [107]. One of the most common autofluorescence-based tests to directly observe oral mucosa self-fluorescence is VELscope (Visual Enhanced Light scope). Many studies reported low specificity values on subjects with OPMDs or OSCC, suggesting possible VELscope^®^ limitations [76,79]. Kaur et al. tried to increase the specificity of VELscope^®^ for OPMD and OSCC detection by combining this tool with additional diagnostic procedures such as salivary protoporphyrin IX levels [72]. Overall, light-based devices are intended for use as an adjunct to a clinical examination and not as a stand-alone diagnostic tool because of their low specificity values, and this seems more realistic considering the high number of individuals included in the studies.

Raman spectroscopy (RS) is a promising non-invasive diagnostic tool for oral cancer and precancer. This technique sheds light on a sample’s molecular composition by analysing how light interacts with its molecules. Raman spectroscopy relies on a phenomenon called Raman scattering. When a laser beam interacts with a molecule, the light can be scattered differently. In a small percentage of cases, the scattered light changes its energy (wavelength). This energy shift provides a unique fingerprint of the vibrational modes within the molecules, revealing their chemical identity and structure [24,108]. By directing a laser beam at oral tissue and analysing the scattered light, RS can potentially identify characteristic changes in the molecular makeup associated with cancer or precancer conditions. RS offers a clear benefit over other optical methods, in that it can provide details about the molecular nature and structure of live tissue. Behl et al. successfully demonstrated, in the pilot study, that RS has the ability to monitor patients with dysplasia over time, reducing the need for multiple biopsies [109]. Overall, RS is being investigated as a diagnostic tool for characterising cancer cells and early malignant changes and distinguishing these cells from normal cells [91,92,96]. Brindha et al. employed RS in the characterisation of the metabolites of human urine in normal subjects and oral cancer patients to discriminate [90]. The weak signals generated by the Raman effect and the widespread overlap of Raman bands due to different biological elements pose serious issues with the use of RS applications, making it challenging to accurately identify specific components. Biomedical samples can provide a strong fluorescence background that can entirely mask the real Raman signals. 

Fluorescence spectroscopy (FS) using a fiber-optic probe interrogation provides an evaluation of a small tissue volume, especially when considering the excitation wavelength in the violet spectrum. Francisco AL et al., using two lasers as excitation light sources at 406 nm and 532 nm to screen 115 subjects, found that the 406 nm excitation provided more tissue information concerning malignant characteristics, even though 532 nm could potentially interrogate a deeper tissue layer [71]. Interestingly, a pilot study has been carried out to check the feasibility of discriminating the saliva of normal individuals and oral cancer patients based on FS at 405 nm excitation using a spectrofluorometer of model Fluoromax-2 with high sensitivity and specificity values [69]. Yuvaraj et al., through this pilot study, posed a first step to extend this simple technique for the mass screening of oral cancer and other cancers [69].

Reflectance optical spectroscopy (ROS) has been used in different studies for the diagnosis of suspicious lesions in several organs, including three studies using the Identafi^®^ device for the screening of suspicious oral lesions [74,94,97]. Beyond structural epithelium changes, neoplasia progression also involves angiogenesis [110]. Tissue reflectance setting in a multispectral device (Identafi^®^) is based on the premise of detecting changes in angiogenesis with green–amber light (545 nm wavelength) illumination. Messadi et al., in their pilot study, correlated the ability of tissue reflectance to detect vascular changes clinically with histological vascularity in various oral lesions [74]. Sharma et al., in a prospective study, found that Identafi^®^ is more sensitive in detecting true positives, whereas toluidine blue is highly specific in ruling out true negative cases [97].

Elastic scattering spectroscopy (ESS) is a promising non-invasive tool applicable for the diagnosis and assessment of tissue pathology in situ, mediated by fiber-optic probes [24]. The ESS method detects microchanges at the subcellular level, and indeed healthy and pathological tissues can generate different spectral signatures as a result of changes in nuclear size, chromatin granularity, organelle sizes and densities, and other sub-cellular features [24]. Optical tissue assessment mediated by ESS technology has shown promising results to support its use in the diagnosis of skin cancer, breast cancer, and colorectal polyps [111,112]. Grillone et al. evaluated the potential application of ESS patterns to distinguish benign, dysplastic, and malignant tissue in the surgical margin of 34 oral lesions, and they obtained a sensitivity value >80% and a moderate specificity value around 80% [95]. Sircan-Kucuksayan et al. obtained a higher specificity value (>90%) in a pilot study, by applying elastic light single-scattering spectroscopy (ELSSS) to a cohort of 47 individuals [98]. However, this study also highlighted the limitations of the ELSSS system. One disadvantage is the probe’s fiber diameter, which is 100 μm; consequently, scanning the entire surface of the lesion requires a lot of time. Another limitation of ELSSS is related to the incorrect classification of low-grade dysplasia as malignant or benign. 

Electrical impedance spectroscopy (EIS) represents a novel and exciting approach for the non-invasive detection of oral cancer and precancer. This technique utilizes the characterization of the intrinsic dielectric properties of cells in tissues to differentiate between healthy and diseased states. EIS analysis measures tissue impedance (due to extracellular fluid) and capacitance (due to cell membranes) by recording a voltage drop in the applied current. Cancerous tissue has been shown to have different electrical properties compared to normal tissue [113]. The main advantage of EIS is its ability to measure cell dynamics in real time. EIS relies on cell polarization generated by an electric field and the interaction of ions along the cell surface [113]. A study demonstrates that EIS can be easily applied to differentiate between the normal oral epithelium and cancer, and it is more important to distinguish between high-risk and low-risk lesions [89]. This technique has been applied as a diagnostic screening tool for the detection of cervical dysplasia and early cervical cancer, skin cancer, and colorectal cancer [114,115,116]. Murdoch et al. applied the EIS technique to an oral district to screen 47 individuals with suspicious lesions [89]. By analysing the electrical properties of tissues, EIS offers a unique perspective on cellular health. However, further research is needed to validate the accuracy of EIS for diagnosing oral cancer in various clinical settings. Developing standardized protocols for EIS measurements and data analysis is crucial for reliable interpretation. 

### 3.5. What Is the Ease of Use for Each Class of Tools? Do They Require Specialised Training for Healthcare Professionals?

Overall, vital staining offers a valuable, cheap test for dentists and healthcare professionals to identify potentially precancerous and cancerous lesions in the oral cavity. For these reasons, vital staining is particularly useful for the detection of cancer in developing countries [36,41].

Among oral brush cytology applications, the conventional method is a simple, well-tolerated, minimally invasive, and relatively painless diagnostic technique for harvesting representative cells of oral mucosal layers. For many years, the most widely used conventional tools to collect oral smears were a cotton-tipped applicator, a wooden tongue depressor, and a metal spatula. The most recent brush is the custom-designed Orcellex^®^ brush, which permits the sampling of representative cells from all layers of the oral mucosal epithelia [59,61,62,65,66]. The collected cell material can be used for diagnostic and research applications like liquid-based cytology (LBC) and PCR. LBC is also easy to perform, relatively painless, and well-accepted by patients. In any case, it was reported that 69.0% and 29.0% of patients feel discomfort and pain, respectively, with LBC [106].

It is claimed that light-based technologies can provide easy management through their ability to identify abnormal oral lesions suspicious for pathology, including precancerous and cancerous cells that may be difficult to see during a regular visual examination [27]. Light-based technologies are non-invasive and non-toxic techniques, and so their implementation as a screening aid would be desirable [28]. However, to accomplish this, the variability among users needs to be sufficiently low so that the device can be used reliably. False negatives may be mitigated by experience at specialty clinics, but in the hands of a general dentist, this is likely to result in a misdiagnosis [33].

Among all classes of tools described here, spectroscopy-based technologies imply the pairing of spectroscopy instruments with hand devices to accomplish data and detect abnormalities, respectively. For example, an EIS device consists of a handheld unit, a base station for downloading data to a laptop, and a single-use sheath covering the snout of the handheld unit. During the diagnostic session, two electrodes were placed gently on the lesions or normal oral mucosa to transfer impedance measurements, and data were recorded in real time and instantly downloaded to a computer for examination [89]. The ESS system includes a pulsed xenon arc lamp (wavelength range is 300–900 nm) as the light source and two fiber-optics (one is used to send light into the tissue and the other to gather dispersed light) [24]. Francisco AL et al. used a fluorescence spectroscopy system composed of two lasers as excitation light sources at 406 nm and 532 nm, an interrogation probe, and a portable spectrophotometer, USB 2000, to screen 115 subjects [71]. In the current scenario, each modality requires different light sources and spectrometers, which makes spectroscopy-based technologies expensive. Overall, spectroscopy-based techniques require a certain mastery of the instrument, and data interpretation can result in complications for those unfamiliar with them. Four factors have been driving instrument development recently: multimodality, instrument sensitivity, size reduction, and clinical acceptance [26,34,115]. All of these prospects for advancement are still open, especially because a doctor will be the end user [115]. In point-of-care diagnostics, combining a portable handheld device with advanced machine-learning models that can offer a real-time output in a language that clinicians can comprehend, with a clear approach, which is necessary [108,113]. In addition, sufficient training regimens must be developed based on global spectroscopic clinical studies that are now underway.

### 3.6. What Is the Individual Non-Invasive Method’s Diagnostic Accuracy (Sensitivity and Specificity)?

**Figure 3:**
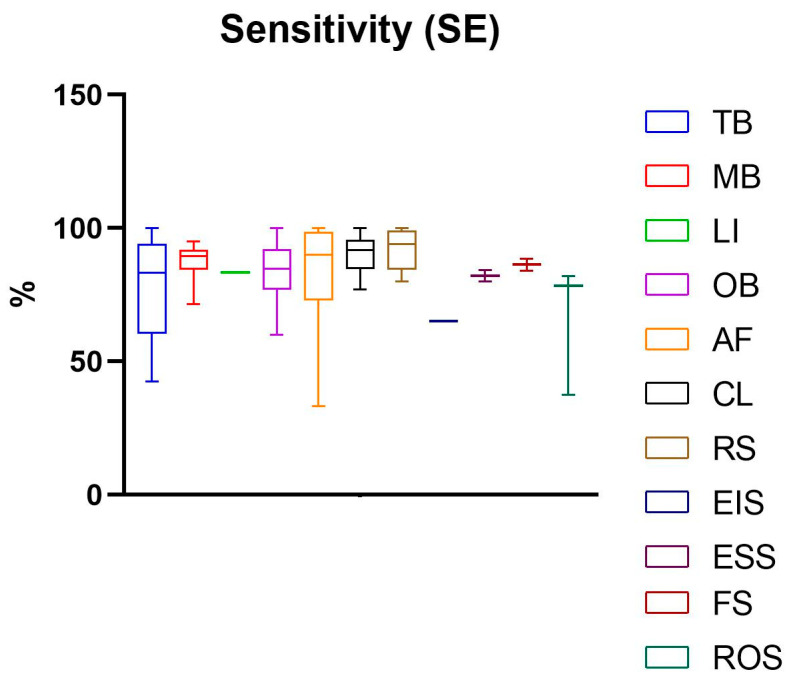
Sensitivity with the relative standard deviation of non-invasive visual diagnostic tools. This graph has been built including values reported in each study. The mean and standard deviations were then calculated.

**Figure 4:**
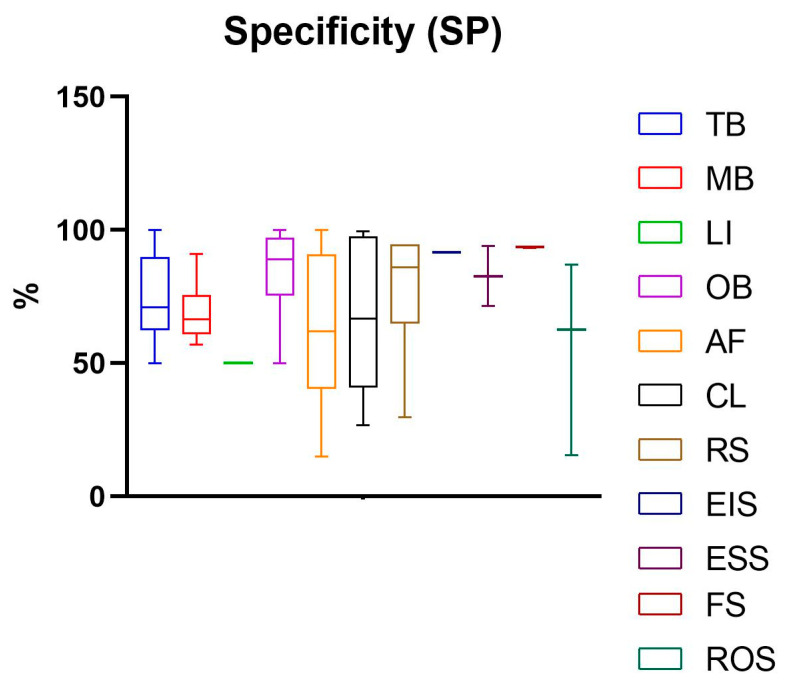
Specificity with the relative standard deviation of non-invasive visual diagnostic tools. This graph has been built including the values reported in each study. The mean and standard deviations were then calculated.

The development and validation of non-invasive diagnostic tools for oral cancer and precancer hold immense promise for improving early detection and patient outcomes. These tools have the potential to reduce the mortality and morbidity associated with oral cancer, particularly in high-risk populations. As research in this area continues to advance, non-invasive diagnostic tools are expected to play an increasingly important role in the fight against oral cancer. The principles of the functioning of non-invasive visual diagnostic tools are very different. Such a great diversity may partly explain the impressive discrepancy of results obtained in the studies analysed. Sensitivity (SE), specificity (SP), positive predictive value (PPV), and negative predictive value (NPV) are common parameters used to assess the performance of a test [117]. SE quantifies the ability of a test to identify true positives for the outcome, while SP quantifies the ability of a test to identify true negatives. Sensitivity and specificity are inversely related—while one grows, the other decreases—but they are considered stable, whereas PPV and NPV can be subjected to variations with pre-test probability that can be derived either from clinician experience or research evidence [117]. For this reason, in this systemic review, only SE and SP have been taken into account to drive the conclusions (Figure 3 and Figure 4).

The vital staining class includes TB, MB, and LI methods. Among six studies evaluating TB, the mean of SE resulted in 77.26% (SD 19.67%), ranging from 42.40% to 100%, while the mean of SP was 74.37% (SD 15.67%) (ranging from 50% to 100%). Among six studies evaluating MB, the mean of SE was 87.47% (SD 8.17%), ranging from 71.4% to 95%. In contrast, MB specificity was less than SE, obtaining a mean value of 68.97% (SD 11.57%), and ranging from 57.10% to 91.00%. Among two studies evaluating LI, the mean values of SE and SP were 83.3% (SD 0) and 50%, respectively. 

The mean value of SE measurements of these three vital stainings was higher than 80% for MB and LI and around 80% for TB; however, the mean value of SP was lower than 80%, indicating that these methods produce a high number of false positives and may incorrectly identify disease in healthy individuals. In particular, Lugol’s iodine displayed a very low SP value of 50%, and this might be due to the presence of some benign lesions that might show reduced iodine uptake, leading to false positives [48,51].

Oral brush includes 18 papers that address both conventional and liquid-based cytology. Overall, the mean SE of these tests resulted in 84.84% (SD 11.28%), ranging from 60% to 100%. Its average specificity was 85.57% (SD 13.55%) (ranging from 50% to 100%). According to reports, in the present study, the mean sensitivity and specificity values were 84.85% and 89.56% for OBC and 80.51% and 64.58% for LBC.

Light-based technologies featured in 21 original articles. Among seven studies evaluating CL, the mean of SE was 90.48% (SD 7.56%) (ranging from 77.10% to 100%); however, the mean of SP was found to be lower than SE, with a value of 66.60% (SD 28.47%) (ranging from 26.80% to 99.60).

In a comparative study, Vashisht et al. compared ViziLite^®^ and TB screening ability in 60 patients. The results revealed that ViziLite^®^ exhibited a diagnostic sensitivity of 95.45% and a specificity of 84.6% and was able to detect early epithelial dysplasia in a high-risk patient with a clinically normal oral mucosa, whereas TB displayed a sensitivity and specificity of 86,36% and 76,9%, respectively [86]. In a cross-sectional study, Chaudhry et al. compared the ViziLite^®^ with TB to assess their clinical usefulness in identifying oral lesions and found that toluidine blue was more effective in identifying the more severe grades of dysplasia, and thus it effectively discriminated high-risk from low-risk lesions [84]. In a comparative study, Shukla et al. found that the ViziLite^®^ chemiluminescence test had higher sensitivity (90%) than the toluidine blue test (63.33%). However, both tests had poor specificity (50%). In order to reduce false positives, the new ViziLite^®^ Plus test combines the diagnostic capabilities of chemiluminescence examination with toluidine blue staining. The results of a study on 40 subjects indicated that the ViziLite^®^ Plus test emerged as the best method for assessing the size, shape, and borders of premalignant lesions. The combination of chemiluminescence and toluidine blue seems to increase sensitivity and specificity by 100% and 97.3%, respectively [83]. Ibrahim et al. evaluated the effectiveness of the chemiluminescence-based test Microlux/DL with and without toluidine blue in screening for potentially malignant and malignant oral lesions. In this comparative study, the results indicated that Microlux/DL is a promising screening device compared to COE, but it exhibits poor specificity (32.4%) compared to the histopathological examination of biopsy specimens, which remains the gold standard. Furthermore, adding toluidine blue dye did not improve the effectiveness of the Microlux/DL system [87]. Swanthi et al. demonstrated that the combination of chemiluminescence-based tool Microlux/DL with toluidine blue had better diagnostic efficiency in detecting dysplasia in tobacco-associated oral lesions when compared to the group of Microlux/DL with Lugol’s iodine [88]. Data concerning the values of SE and SP of AF were reported in 13 studies. The most common AF tool was VELscope. The mean of AF sensibility was 83.32% (SD 18.88%), ranging from 33.30% to 100%, while the mean value for SP was 62.39% (SD 20.04%), ranging from 15% to 100%. Bhatia et al. found that the sensitivity of VELscope™ when compared with the soft gold standard COE was 64.0%, while the sensitivity for a combined examination was greater than either examination alone, at 73.9%. This was associated with only a small drop in specificity, from 99.0% for COE to 97.9% for combined findings [70]. Recently, other authors have obtained higher sensitivity and specificity values by performing fluorescence visualization, applying different light-emitting instruments such as ORALOOK^®^, IllumiScan^®^, and EVINCE^®^ [75,80,82].

Spectroscopy-based technology includes 13 original articles. The six studies evaluating RS showed a mean value of SE 92.15% (SD 7.68) (ranging from 80% to 100%) and a mean value of SP of 77.93% (SD 24.51%) (ranging from 29.70% to 94.40%). Regarding FS, two papers were evaluated that reported an SE of 86.30% (SD 3.11%) and SP of 93.50% (SD 0.42%). Among the three papers that considered ROS, SE 65.97% (SD 24.72%) ranging from 37.50% to 82% and SP 54.97% (SD 36.39%) ranging from 15.4% to 87% were reported. Only one study was found for the EIS tool, and the reported percentages of SE and SP were 65.20% and 91.70%, respectively. The evaluation of two studies concerning ESS reported a mean SE of 82.10% (SD 2.97%) and SP of 82.70% (SD 15.98%).

Overall, the SE and SP values reported in the studies described here are poorly acceptable for oncologic diagnosis, but they seem more realistic because standard deviations are low.

### 3.7. What Are the Future Directions for Researching Non-Invasive Diagnostic Tools for Oral Cancer?

The future of oral cancer diagnosis can rely on combining different techniques to overcome the shortcomings of a single technique. The combination of various methods could potentially lead to new diagnostic approaches and increase diagnostic accuracy, which is the major limitation of non-invasive tools. For example, the sensitivity and spatial resolution of conventional imaging methods are insufficient. A possible solution to these limitations could lie in multiscale and multimodal imaging techniques that combine macroscopic biochemical imaging with morphological subcellular imaging techniques, which could also lead to new avenues in clinical research. Another possible improvement could be achieved by implementing new technologies such as artificial intelligence (AI). AI is an advancement that holds promise for significantly improving the effectiveness of diagnosing oral cancer. AI systems, trained on massive datasets, can analyse images, patient history, and even genetics to predict oral cancer. Potentially, the future of oral cancer diagnosis lies in the miniaturisation of devices that can improve the attractiveness of the technology from a financial and economic point of view, in addition to its design and space-saving. Lab-on-a-Chip (LOC) technology can miniaturise biological molecule detection onto a tiny chip. In particular, biosensor-integrated devices such as EIS might be suitable for LOC technology. This highlights the importance of further research and development to refine and improve these tools to better identify pre-cancerous lesions and oral cancer. Finally, there is a growing body of evidence suggesting a strong link between oral microbiota dysbiosis and the development of oral cancer [118]. The field of oral cancer diagnosis and understanding of the oral microbiota is rapidly evolving, with a strong focus on non-invasive methods that can assess both oral cancer and oral microbiota, providing valuable information for early detection and risk assessment and potentially developing novel therapeutic strategies. Potentially, salivary microbiota might be considered as a non-invasive diagnostic tool for tracking alterations in human physiology, as well as the saliva biomarkers already used [119,120]. Beyond saliva, dental plaque and oral rinse represent valuable samples for studying the oral microbiota, and clinicians can easily collect them. On the other hand, the technologies used to analyse microbial composition, such as DNA sequencing or bacterial metabolic products, such as metabolomics, present some challenges.

## 4. Discussion

This study can be considered useful for the evaluation of the optimal techniques to examine suspicious lesions, as well as on the basis of the qualitative and quantitative values of important properties such as sensitivity and specificity. Several methods for oral cancer diagnosis were not included in this systematic review because each of those diagnostic methods is based on a visual oral examination, which makes the methods easy to apply and generates fast results. The various non-invasive methods used in recent years (2014–2024) for the early screening of oral cancer, including vital staining, oral brush, light-based tools, and spectroscopy-based tools, have been summarized. All these methods possess the advantages of being minimally invasive, painless, fast, and economical diagnostic techniques, making them comfortable and patient-friendly procedures. However, each method has its own disadvantages and necessitates further research and breakthroughs before these tools can replace biopsies for the early diagnosis of oral cancer. Spectroscopy-based methods offer different dimensions and perspectives of the oral cavity; for this reason, they have great clinical application prospects. However, developing robust algorithms for accurate interpretation remains an ongoing challenge. Overall, the quantity of included studies for each diagnostic tool was satisfactory. However, the number of studies concerning LI, EIS, and ESS was limited to two, one, and two, respectively, within the 2014–2024 timeframe. The included studies exhibited heterogeneity in terms of design, with only three randomized controlled trials (RCTs). The remaining studies were cohort, cross-sectional, and case–control studies, encompassing sample sizes ranging from 10 to 877 participants. Among the 58 non-randomized studies, the methodological quality, assessed using the NOS, was high for 8, moderate for 47, and low for 3 studies. Additionally, the restriction to English-language articles might have led to the exclusion of relevant studies published in other languages.

## 5. Conclusions

Non-invasive diagnostic tools for oral cancer offer potential benefits for early detection. However, current methods exhibit limitations in accuracy and reliability. Vital staining using dyes like TB, MB, and LI is the simplest and cheapest method. While sensitive in identifying abnormal tissue, it often produces false positives, incorrectly flagging healthy tissue as potentially cancerous. Oral brush cytology (OBC) collects cells for microscopic examination, offering a minimally invasive approach. However, its sensitivity and specificity are moderate, requiring further evaluation. Light-based technologies such as CL and AF can detect tissue abnormalities but suffer from low specificity and high rates of false positives and negatives. Spectroscopy-based technologies, including RS, FS, ROS, ESS, and EIS, analyse light or electric interaction with tissue to identify molecular changes. These methods require specialised equipment and expertise, and their diagnostic accuracy remains variable. Despite advancements, incisional biopsy continues to be the gold standard for the definitive diagnosis of oral cancer and precancerous lesions. Further research and development are essential to improve the sensitivity, specificity, and reliability of non-invasive tools for widespread clinical application.

## Figures and Tables

**Table 1:** List of review articles included in the full-text analysis to search for further original articles.

Reference	Author(s)	Year	Journal Abbreviation	Title	Summary of Main Findings
[14]	Kong K, Kendall C, Stone N, Notingher I.	2015	*Adv Drug Deliv Rev.*	Raman spectroscopy for medical diagnostics-From in-vitro biofluid assays to in-vivo cancer detection.	Metallic nanoparticles can be used to enhance the Raman signals and optimised fibre-optic Raman probes can be used for real-time in vivo single-point measurements, while multimodal integration with other optical techniques can guide the Raman measurements to increase the acquisition speed and spatial accuracy of diagnosis.
[15]	Balasubramaniam AM, Sriraman R, Sindhuja P, Mohideen K, Parameswar RA, Muhamed Haris KT.	2015	*J Pharm Bioallied Sci.*	Autofluorescence based diagnostic techniques for oral cancer.	Autofluorescence-based diagnostic techniques are rapidly emerging as a powerful tool.
[16]	Tiwari L, Kujan O, Farah CS.	2020	*Oral Dis.*	Optical fluorescence imaging in oral cancer and potentially malignant disorders: A systematic review.	Devices utilising optical fluorescence imaging are viewed strictly as clinical adjuncts and not specifically as diagnostic devices.
[17]	Lingen MW, Tampi MP, Urquhart O, Abt E, Agrawal N, Chaturvedi AK, Cohen E, D’Souza G, Gurenlian J, Kalmar JR, Kerr AR, Lambert PM, Patton LL, Sollecito TP, Truelove E, Banfield L, Carrasco-Labra A.	2017	*J Am Dent Assoc.*	Adjuncts for the evaluation of potentially malignant disorders in the oral cavity: Diagnostic test accuracy systematic review and meta-analysis-a report of the American Dental Association.	Cytologic testing appears to be the most accurate adjunct. The main concerns are the high rate of false-positive results and serious issues of risk of bias and indirectness of the evidence.
[18]	Mascitti M, Orsini G, Tosco V, Monterubbianesi R, Balercia A, Putignano A, Procaccini M, Santarelli A.	2018	*Front Physiol.*	An Overview on Current Non-invasive Diagnostic Devices in Oral Oncology.	The Light-Based Detection Systems showed great potential for screening and monitoring oral lesions, but there are several factors that hinder an extensive use of these devices.
[19]	Ravikumar R, Sunil BK, Abdulaziz AA, Sree LC, Darshan DD, Fawad J, Ihtesham UR.	2016	*Applied Spectroscopy Reviews.*	Applications of Raman spectroscopy in dentistry part II: Soft tissue analysis.	Raman spectroscopy is used to identify the molecular structures and their components to give substantial information about the chemical structure properties of these molecules.
[20]	Shashidara R, Sreeshyla HS, Sudheendra US.	2014	*J Cancer Res Ther.*	Chemiluminescence: a diagnostic adjunct in oral precancer and cancer: a review.	Chemiluminescence is one of the newly developed adjuncts.
[21]	Wang S, Yang M, Li R, Bai J.	2023	*Eur J Med Res.*	Current advances in non-invasive methods for the diagnosis of oral squamous cell carcinoma: a review.	Liquid biopsy biomarkers, including novel microbiome components, noncoding RNAs, extracellular vesicles, and circulating tumour DNA, have demonstrated encouraging clinical outcomes in early OSCC detection.
[22]	Bisht SP, Mishra P, Yadav D, Rawal R, Mercado-Shekhar KP.	2021	*Prog. Biomed. Eng.*	Current and emerging techniques for oral cancer screening and diagnosis: a review.	Few studies have tested emerging techniques in the context of oral cancer.
[23]	Gupta S, Jawanda MK, Madhushankari GS.	2020	*J Oral Biol Craniofac Res.*	Current challenges and the diagnostic pitfalls in the grading of epithelial dysplasia in oral potentially malignant disorders: A review.	Robust research on the predictive value, relevance, and applicability and large-scale studies are still required to discover a reliable and reproducible method for the grading of OED.
[24]	Esam O.	2015	*Head Face Med.*	Current concepts and future of noninvasive procedures for diagnosing oral squamous cell carcinoma: a systematic review.	Advances in technologies for saliva-based oral diagnosis and optical biopsy are promising pathways for the future development of more effective non-invasive methods for diagnosing OSCC.
[25]	Su YF, Chen YJ, Tsai FT, Li WC, Hsu ML, Wang DH, Yang CC.	2021	*Diagnostics (Basel).*	Current Insights into Oral Cancer Diagnostics.	The rapid development of novel biomarkers, electronic systems, and artificial intelligence may help to develop a new era in which OPMD and oral cancer are detected at an early stage.
[26]	Strome A, Kossatz S, Zanoni DK, Rajadhyaksha M, Patel S, Reiner T.	2018	*Mol Imaging.*	Current Practice and Emerging Molecular Imaging Technologies in Oral Cancer Screening.	Imaging agents that are easy to use, inexpensive, non-invasive, and specific can be utilized to increase the number of patients who are screened and monitored in a variety of different environments.
[27]	Awan KH, Patil S.	2015	*J Contemp Dent Pract.*	Efficacy of Autofluorescence Imaging as an Adjunctive Technique for Examination and Detection of Oral Potentially Malignant Disorders: A Systematic Review.	VELscope shows high sensitivity values in detecting oral premalignant and malignant lesions. However, it has an inability to discriminate dysplasia cases from nondysplasia cases.
[28]	Nagi R, Reddy-Kantharaj YB, Rakesh N, Janardhan-Reddy S, Sahu S.	2016	*Med Oral Patol Oral Cir Bucal.*	Efficacy of light based detection systems for early detection of oral cancer and oral potentially malignant disorders: Systematic review.	Light-Based Detection Systems are a simple, non-invasive test of the oral mucosa but are suited for clinicians with sufficient experience and training.
[29]	Kim DH, Kim SW, Hwang SH.	2022	*Braz J Otorhinolaryngol.*	Efficacy of non-invasive diagnostic methods in the diagnosis and screening of oral cancer and precancer.	Narrow banding imaging has superiority in terms of sensitivity and negative predictive value compared with the other tested agents.
[30]	Kim DH, Song EA, Kim SW, Hwang SH.	2021	*Clin Otolaryngol.*	Efficacy of toluidine blue in the diagnosis and screening of oral cancer and pre-cancer: A systematic review and meta-analysis.	TB should be combined with chemiluminescence or other diagnostic tools.
[31]	Mazur M, Ndokaj A, Venugopal DC, Roberto M, Albu C, Jedliński M, Tomao S, Vozza I, Trybek G, Ottolenghi L, Guerra F.	2021	*Int J Environ Res Public Health.*	In Vivo Imaging-Based Techniques for Early Diagnosis of Oral Potentially Malignant Disorders-Systematic Review and Meta-Analysis.	In vivo imaging-based techniques based on assessing oral images cannot replace the biopsy.
[32]	Chhabra N, Chhabra S, Sapra N.	2015	*J Maxillofac Oral Surg.*	Diagnostic modalities for squamous cell carcinoma: an extensive review of literature-considering toluidine blue as a useful adjunct.	Toluidine blue is a screening modality and not a diagnostic procedure like biopsy.
[33]	Yang EC, Tan MT, Schwarz RA, Richards-Kortum RR, Gillenwater AM, Vigneswaran N.	2018	*Oral Surg Oral Med Oral Pathol Oral Radiol.*	Noninvasive diagnostic adjuncts for the evaluation of potentially premalignant oral epithelial lesions: current limitations and future directions.	In vivo microscopy technologies allow clinicians to visualize many of the same microscopic features used for histopathologic assessment at the point of care.
[34]	Romano A, Di Stasio D, Petruzzi M, Fiori F, Lajolo C, Santarelli A, Lucchese A, Serpico R, Contaldo M.	2021	*Cancers (Basel).*	Noninvasive Imaging Methods to Improve the Diagnosis of Oral Carcinoma and Its Precursors: State of the Art and Proposal of a Three-Step Diagnostic Process.	An ideal three-step diagnostic procedure can make the diagnostic path faster, better, and more accurate.
[35]	Borkar S, Reche A, Paul P, Deshpande A, Deshpande M.	2023	*Cureus.*	Noninvasive Technique for the Screening and Diagnosis of Oral Squamous Cell Carcinoma.	Optical coherence tomography and high-frequency ultrasound are considered to be beneficial, particularly for assessing the dimensions of tumours before surgery.
[36]	Liu D, Zhao X, Zeng X, Dan H, Chen Q.	2016	*Tohoku J Exp Med.*	Non-Invasive Techniques for Detection and Diagnosis of Oral Potentially Malignant Disorders.	Vital staining with a solution that can be used as a mouth rinse, light-based detection systems, optical diagnostic technologies that employ returned optical signals to reflect structural and morphological changes within tissues, and salivary biomarkers.
[37]	Singh SP, Ibrahim O, Byrne HJ, Mikkonen JW, Koistinen AP, Kullaa AM, Lyng FM.	2016	*Head Neck.*	Recent advances in optical diagnosis of oral cancers: Review and future perspectives.	The prospective adaptation of optical spectroscopy methods for routine clinical diagnosis would decrease the number of follow-up clinic visits and patient anxiety by minimizing waiting times for histopathological diagnosis.
[38]	Rashid A, Warnakulasuriya S.	2015	*J Oral Pathol Med.*	The use of light-based (optical) detection systems as adjuncts in the detection of oral cancer and oral potentially malignant disorders: a systematic review.	There is limited evidence for the use of light-based detection systems in primary care, and these tools are better suited to specialist clinics in which there is a higher prevalence of disease and where experienced clinicians may better discriminate between benign and malignant lesions.
[39]	H Alsarraf A, Kujan O, Farah CS.	2018	*J Oral Pathol Med.*	The utility of oral brush cytology in the early detection of oral cancer and oral potentially malignant disorders: A systematic review.	There is a need for well-designed clinical studies to assess the accuracy of oral brush cytology utilizing validated cytological assessment criteria for the diagnosis and prediction of OPMDs.

**Table 2:** List of studies identified using keywords, inclusion criteria, and review checking. Data extracted from each study included authors and publication year, typology of the study, diagnostic tool analysed, number of lesions evaluated, number of patients included, number of pre-cancer lesions, number of cancer lesions, SE, SP, PPV, NPV, and the main conclusions of the study.

Authors, Year, Reference	Typology of Study	Diagnostic Tools	Number of Lesions	Patient Population Recruited	Pre-Cancer Lesions	Cancer Lesions	SE (%)	SP (%)	PPV (%)	NPV (%)	Main Conclusions
Singh D et al., 2015 [41]	Cohort study	TB	45	45	-	45	97.80%	100%	100%	80%	TB assists in the choice of biopsy sites.
Soman C et al., 2016 [42]	RCT	MB	60	120	41	16	90.60%	57.10%	70.70%	84.20%	MB has less toxic effects and it is a valuable chairside diagnostic tool in the early detection of OPMD.
Lejoy A et al., 2016 [43]	Case–control study	MB	75	125	70	5	95%	70%	91%	80%	MB shows low toxicity and it is cheaper than TB.
Vijayakumar V et al., 2017 [44]	Cohort study	TB	55	55	7	34	92.60%	67.90%	-	-	TB is useful in large-scale oral screening of high-risk patients.
Parakh MK et al., 2017 [45]	Case–control study	TB	17	500	-	16	88.89%	74.19%	50%	97.83%	TB is a useful tool for identifying biopsy sites in potentially malignant lesions.
Nethan ST et al., 2018 [46]	Cross-sectional study	MB	50	50	33	-	71.4%	62.5%	90.9%	29.4%	MB dye is an efficacious diagnostic adjunct and a suitable alternative to TB.
Gupta M et al., 2019 [47]	Case–control study	MB	50	50	20	18	89%	91%	97%	73%	MB is a useful diagnostic adjunct in a large, community-based oral cancer screening program for high-risk individuals.
Ali Channa S et al., 2019 [48]	Cohort study	MB vs. LI	60	60	60	-	89.4% (MB) 83.3% (LI)	66.6% (MB) 50% (LI)	-	-	MB and LI are useful tools for the early diagnosis of OPMD and OSCC but specificity is not high.
Algadi HH et al., 2020 [49]	Cohort study	TB	140	30	-	6	100%	94%	42.90%	100%	TB is able to identify positive mucosal tumour margins with no false negatives and a slightly lower specificity.
Jayasinghe RD et al., 2020 [50]	Cohort study	TB	65	65	23	5	68.30%	63.10%	80%	48%	TB might be a potential adjunct diagnostic aid in identifying high-risk oral cancers.
Iqbal W et al., 2023 [51]	Cross-sectional study	LI vs. MB	60	60	60	-	83.3% (LI) vs. 89.4% (MB)	50% (LI) vs. 66.6% (MB)	-	-	LI and MB are easy screening tools for the early diagnosis of malignancy.
Ma JM et al., 2014 [52]	Cohort study	OB	52	52	47	-	86.36%	90%	86.36%	90%	OB is a useful method for monitoring potentially malignant oral disorders.
Trakroo A et al., 2014 [53]	Case–control study	OB	27	50	27	-	84.37%	88.89%	93.10%	76.19%	OB is suitable in population screening programs and for pre- and post-treatment observation of OPMD and OSCC.
Gupta S et al., 2014 [54]	Case–control study	OB vs. cytology	1099	877	225	-	81.69% (OB) vs. 48.57% (citology)	68.42% (OB) vs. 86.48% (cytology)	-	-	OB shows higher sensibility and specificity than cytology.
Jajodia E et al., 2016 [55]	Cross-sectional study	OB liquid	-	48	13	32	75%	50%	96.80%	9.10%	OB is a useful screening modality due to reducing the likelihood of false positives and false negatives.
Kaur M et al., 2016 [56]	Cohort study	OB	96	100	6	36	83.30%	95.80%	95.20%	85.20%	OB is a method for analysing suspicious oral lesions with acceptable sensitivity and specificity.
Nanayakkara PG et al., 2016 [57]	Cohort study	OB	192	192	116	76	89.58%	100%	100%	80.77%	OB is a useful screening instrument for early diagnosis of suspicious oral lesions and could contribute to improved oral cancer prognosis.
Sekine J et al., 2017 [58]	Case–control study	OB	423	-	-	153	77.80%	83.90%	81%	81.10%	OB is useful to identify borderline lesions.
Goodson ML et al., 2017 [59]	Case–control study	OB (Orcellex)	145	310	131	14	60%	99%	67%	99%	OB provides reliable diagnoses consistent with conventional histopathology and is less invasive and appropriate for the long-term monitoring of patients.
Remmerbach TW et al., 2017 [60]	Cohort study	OB liquid	113	113	13	64	97.53%	68.75%	88.76%	91.67%	OB can not substitute the scalpel biopsies but it provides a quick and reliable screening tool to identify OSCC at an early stage.
Alsarraf A et al., 2018 [61]	Case–control study	OB (Orcellex)	134	86	90	11	75%	76%	76%	75%	Orcellex^®^ OB can be used as an adjunct for the early detection of oral cancer.
Pandey P et al., 2018 [62]	Cohort study	OB (Orcellex)	110	110	72	38	100%	97.5%	93.9%	100%	OB is a useful tool to perform an initial screening.
Kokubun K et al., 2023 [63]	Case–control study	OB liquid	251	653	-	82	69%	75%	38%	75%	OB diagnosis of OSCC is occasionally inconsistent with the histological diagnosis.
Velleuer E et al., 2020 [64]	Cohort study	OB	1233	713	737	-	97.7%	84.5%	45.4%	99.6%	OB appears to identify visible oral, potentially malignant and malignant lesions at an early stage.
Neumann F et al., 2022 [65]	Cross-sectional study	OB (Orcellex)	814	670	725	83	100%	86.5%	43.1%	100%	OB might help dentists in cases of doubt to prevent tumours from expansive growth.
Kujan O et al., 2021 [66]	Cross-sectional study	OB (Orcellex)	72	72	61	10	90.14%	96.17%	-	-	OB provides a minimally invasive adjunct to surgical biopsy.
Castillo P et al., 2022 [67]	Cohort study	OB	-	75	-	24	88%	100%	-	-	OB shows good accuracy for the diagnosis of OSCC before treatment, but its value decreases after treatment.
Idrees M et al., 2022 [68]	Cross-sectional study	OB	284	284	265	19	79.23%	94.81%	-	-	OB is a reliable adjunct to surgical biopsy in the diagnosis of OSCC and OPMD.
Yuvaraj M et al., 2014 [69]	Case–control study	FS Fluoromax (405 nm)	63	123	-	63	84.10%	93.20%	-	-	FS aids in discriminating oral lesions from the normal tissue.
Bhatia N et al., 2014 [70]	Cohort study	COE vs. AF VELscope (400–460 nm)	222	146	222	-	COE 44% vs. VELscope 64%	COE 99% vs. VELscope 97.9%	-	-	VELscope™ can be used in routine general dental practice without compromising patient care.
Francisco AL et al., 2014 [71]	Cohort study	FS (USB2000)	99	115	-	56	88.5%	93.8%	-	-	Excitation at 406 nm was more efficient and can be used as an auxiliary tool in the clinical diagnostic discrimination of OPMD and OSCC.
Kaur J et al., 2015 [72]	Cross-sectional study	AF VELscope (400–450 nm)	85	130	55	25	67%	62%	-	-	VELscope™ is able to discriminate OPMD and OSCC from healthy tissues.
Scheer M et al., 2016 [73]	Cohort study	AF VELscope(400–460 nm)	-	41	27	5	33.30%	88.60%	33.30%	88.60%	VELscope™ does not give additional information for diagnosis in a small group of patients.
Messadi DV et al., 2014 [74]	Cross-sectional study	Identafi (AF 405 nm and ROS 545 nm)	21	21	11	5	ROS 82%	ROS 87%	-	-	ROS clarifies the understanding of microvascular changes in OPMD and OSCC.
Simonato LE et al., 2017 [75]	Case–control study	AF EVINCE (400 nm)	7	15	2	5	100%	46%	22.22%	100%	AF is capable of improving inexperienced professionals’ efficacy for the early detection of oral lesions more prone to be dysplastic.
Ganga RS et al., 2017 [76]	Cross-sectional	AF (VELscope)	200	-	-	25	76%	66.29%	24.36%	95.08%	AF can serve to alleviate patient anxiety regarding suspicious mucosal lesions.
Yamamoto N et al., 2017 [77]	Cross-sectional study	AF VELscope (400–460 nm)	79	62	49	30	90.6%	80.0%	95.1%	66.7%	AF has potential as an auxiliary method for the diagnosis of OPMD.
Cânjău S et al., 2018 [78]	Cross-sectional study	AF VELscope (400–460 nm)	18	18	1	8	94.44%	100%	100%	50%	AF is useful for clinical examination, monitoring oral lesions, and guiding the biopsy.
Amirchaghmaghi M et al., 2018 [79]	Cross-sectional study	AF VELscope (400–460 nm)	45	45	12	9	90%	15%	40%	71%	AF is not capable of distinguishing benign lesions from OPMD and OSCC due to its low specificity.
Simonato LE et al., 2019 [80]	Cohort study	AF (EVINCE, 400 nm)	137	137	32	2	100%	92.40%	-	-	AF has potential to be used as an adjunctive method for the early diagnosis of oral high-risk lesions.
Chiang TE et al., 2019 [81]	Cross-sectional study	AF (Horus UOC 100, 400–460 nm)	68	126	68	-	77.94%	35.42%	63.10%	53.13%	AF aids in the exclusion of the non-OPMD cases.
Morikawa T et al., 2020 [82]	Cross-sectional study	AF (ORALOOK, 422–425 nm)	396	502	123	161	96.80%	48.40%	-	-	AF shows high sensitivity and specificity for the detection of OSCC.
Jain N et al., 2018 [83]	Cross-sectional study	CL (ViziLite Plus) + TB	40	40	40	-	100%	93.7%	100%	75%	CLTB improves the visualization of potentially premalignant lesions.
Chaudhry A et al., 2016 [84]	Cross-sectional study	CL (ViziLite) vs. TB	100	100	100	-	84.84% (CL) vs. 42.40% (TB)	41.17% (CL) vs. 88.23% (TB)	73.68% (CL) vs. 87.50% (TB)	58.33% (CL) vs. 44.11% (TB)	TB is more effective in identifying the more severe grades of dysplasia.
Shukla A et al., 2018 [85]	Cohort study	CL (ViziLite) vs. TB	42	42	28	14	90% (CL) vs. 63.3% (TB)	50% (CL) vs. 50% (TB)	-	-	Both CL and TB can be used as an adjunct to simple, conventional visual examination and in screening procedures for OPMD.
Vashisht N et al., 2014 [86]	Cohort study	CL (ViziLite) vs. TB	50	60	23	-	95.45% (CL) vs. 86.36% (TB)	84.60% (CL) vs. 76.90% (TB)	-	-	CL is relatively reliable in screening OPMD compared to TB.
Ibrahim SS et al., 2014 [87]	RCT	CL (Microlux/DL) vs. COE CL (Microlux/DL) vs. biopsy	-	599	53	-	94.3% (CL) vs. COE 100% (CL) vs. biopsy	99.6% (CL) vs. COE 32.4% (CL) vs. biopsy	96.2% (CL) vs. COE 17.9% (CL) vs. biopsy	99.5% (CL) vs. COE 100% (CL) vs. biopsy	CL is not effective when the diagnostic gold standard remains the histopathological examination of biopsy.
Swathi KV et al., 2021 [88]	RCT	CL (Microlux/DL) + TB vs. CL (Microlux/DL) + LI	85	84	84	-	91.7% (CLLI) vs. 100% (CLTB)	66.7% (CLLI) vs. 60% (CLTB)	84.6% (CLLI) vs. 93.3% (CLTB)	80% (CLLI) vs. 100% (CLTB)	CLTB has a better diagnostic efficiency in OPMD than CLLI.
Murdoch C et al., 2014 [89]	Case–control study	EIS (785 nm)	47	47	27	10	65.20%	91.70%	-	-	EIS aids in identifying the best site for biopsy and for monitoring lesions for disease progression over time.
Elumalai B. et al., 2014 [90]	Case–control study	RS (500–1800 cm^−1^)	93	167	-	93	98.60%	87.10%	-	-	RS could be considered in diagnostic oncology in discriminating cancer patients from healthy subjects.
Krishna H et al., 2014 [91]	Case–control study	RS (785 nm)	515	171	199	316	94.20%	94.40%	-	-	RS has strong potential to provide real-time, non-invasive diagnosis of OSCC and OPMD.
Knipfer C et al., 2014 [92]	Cross-sectional study	RS (770–810 nm)	12	12	-	12	86.10%	94.40%	-	-	RS has been proven to objectively identify OSCC tissue with excellent results.
Guze K et al., 2015 [93]	Cross-sectional study	RS (785 nm)	31	18	4	11	100%	77%	-	-	RS offers the potential to provide point-of-care diagnosis of oral disease using cheap technology.
Lalla Y et al., 2016 [94]	Cohort study	Identafi (AF 405 nm and ROS 545 nm)	231	88	38	2	AF 12.50% ROS 37.5%	AF 85.40%ROS 62.5%	-	-	Identafi produces equivalent visualization of the oral cavity compared with an extraoral white-light source.
Grillone GA et al., 2017 [95]	Cohort study	ELSS (330–660 nm)	34	34	-	27	84.20%	71.40%	69.57%	85.37%	ELSS provides fast, real-time assessment of tissue without the need for pathology expertise.
Malik A et al., 2017 [96]	Cohort study	RS (785 nm)	42	99	28	14	80%	29.7%	-	-	RS is a useful for identification of sites that have higher propensity to progress to carcinomas.
Sharma D et al., 2021 [97]	Cohort study	Identafi (AF 405 nm and ROS 545 nm) vs. TB	63	49	63	-	AF 73% ROS 78.4% vs. TB 51.4%	AF 46.2% ROS 15.4% vs. TB 84.6%	AF 57.6% ROS 56.86% vs. TB 82.6%	AF 46.2% ROS 33.33% vs. TB 84.6%	Identafi’s violet light and green/amber light are more sensitive in detecting true positives.
Sircan-Kucuksayan A et al., 2020 [98]	Cross-sectional study	ESS (350–800 nm)	52	47	-	6	80%	94%	-	-	ELSS may reduce the number of unnecessary biopsies.
Petruzzi M et al., 2014 [99]	Cross-sectional study	AF (VELscope) vs. TB	56	49	19	37	70% (AF) vs. 80% (TB)	57.7% (AF) vs. 61.5% (TB)	65.6% (AF) vs. 70.6% (TB)	62.5% (AF) vs. 72.7% (TB)	AF and TB are both sensitive but not specific in OSCC and OPMD.
Awan KH et al., 2015 [100]	Cohort study	AF (VELscope)vs CL (Vizilite) vs. TB	126	-	126	-	87.1% (AF) vs. 77.1% (CL) vs. 52.9 (TB)	21.4% (AF) vs. 26.8% (CL) vs. 67.9% (TB)	37.8% (AF) vs. 39.5% (CL) vs. 50% (TB)	61.1% (AF) vs. 66.7% (CL) vs. 71.2% (TB)	The accuracy in identifying OPMD is questionable. In combination, the tests yielded better results with improved specificity.
Behl I et al., 2020 [101]	Case–control study	RS (400–1800 cm^−1^) vs. OB	-	40	20	-	94% (OB) vs. 86% (RS)	85% (OB) vs. 85% (RS)	-	-	The adoption of RS to detect degrees of dysplasia is at the “proof of concept” stage, but results are very encouraging.

**Table 3:** List of studies of non-invasive visual diagnostic tools identified through references and grouped into four classes.

Class/Principle	Number of Studies	Type of Diagnostic Tool	References
Vital staining	16	Toluidine blue (TB)	[41,42,43,44,45,46,47,48,49,50,51,84,85,86,99,100]
		Methylene blue (MB)	[41,42,43,44,45,46,47,48,49,50,51,84,85,86,99,100]
		Lugol’s Iodine (LI)	[41,42,43,44,45,46,47,48,49,50,51,84,85,86,99,100]
Oral brush (OB)	18	Oral brush biopsy	[52,53,54,55,56,57,58,59,60,61,62,63,64,65,66,67,68,101]
		Liquid brush cytology	[52,53,54,55,56,57,58,59,60,61,62,63,64,65,66,67,68,101]
Light-based technology	19	Chemiluminescence (CL)	[70,72,73,75,76,77,78,79,80,82,83,84,85,86,87,88,99,100]
		Autofluorescence (AF)	[70,72,73,75,76,77,78,79,80,82,83,84,85,86,87,88,99,100]
Spectroscopy	13	Raman spectroscopy (RS)	[69,71,74,89,90,91,92,93,94,95,96,97,98,101]
		Fluorescence spectroscopy (FS)	[69,71,74,89,90,91,92,93,94,95,96,97,98,101]
		Reflectance optical spectroscopy (ROS)	[69,71,74,89,90,91,92,93,94,95,96,97,98,101]
		Electrical impedance spectroscopy (EIS)	[69,71,74,89,90,91,92,93,94,95,96,97,98,101]
		Elastic scattering spectroscopy (ESS)	[69,71,74,89,90,91,92,93,94,95,96,97,98,101]

## Data Availability

Data are contained within the article and Supplementary Materials.

## Supplementary Materials

The following supporting information can be downloaded at: https://www.mdpi.com/article/10.3390/diagnostics14182033/s1, Supplementary S1: Quality Aspects Rated Using the Newcastle–Ottawa Scale (NOS); Table S1: NOS scale for quality rating of cohort studies.; Table S2: NOS scale for quality rating of cross-sectional studies. Table S3: NOS scale for quality rating of case–control studies. Table S4: NOS for quality rating of all studies.
