# Supplementary material for: The Diagnostic Potential of Non-Invasive Tools for Oral Cancer and Precancer: A Systematic Review
Source: Diagnostics (Basel). 2024 Sep 13;14(18):2033. doi: 10.3390/diagnostics14182033 (PMC11431589; doi:10.3390/diagnostics14182033)
Supplement: Supplementary file 1 [file diagnostics-14-02033-s001.zip › diagnostics-3165225-supplementary.pdf]

ONLINE SUPPLEMENTARY

Supplementary S1: Quality Aspects Rated Using the Newcastle–Ottawa Scale (NOS)

Cohort Studies

Selection (max. four stars):

1) Representativeness of the exposed cohort:

a) Truly representative (**one star**);

b) Somewhat representative (**one star**);

c) Selected group;

d) No description of the derivation of the cohort.

2) Selection of the non-exposed cohort:

a) Drawn from the same community as the exposed cohort (**one star**);

b) Drawn from a different source;

c) No description of the derivation of the non-exposed cohort.

3) Ascertainment of exposure:

a) Secure record (e.g., biopsy) (**one sta**r);

b) Structured interview (**one star**);

c) Written self-report;

d) No description;

e) Other.

4) Demonstration that the outcome of interest was not present at the start of the study:

a) Yes (**one star**);

b) No.

Comparability (max. two stars):

1) Comparability of cohorts on the basis of the design or analysis, controlled for confounders:

a) The study controls for age, sex and marital status (**one star**);

b) Study controls for other factors (tobacco/alcohol/location of lesions) (**one star**);

c) Cohorts are not comparable on the basis of the design or analysis, controlled for confounders.

Outcome (max. three stars):

1) Assessment of outcome:

a) Independent blind assessment (**one sta**r);

b) Record linkage (**one star**);

c) Self-report;

d) No description;

e) Other.

2) Was follow-up long enough for outcomes to occur (>6 months):

a) Yes (**one star**);

b) No.

3) Adequacy of follow-up of cohorts:

a) Complete follow up: all subject accounted for (**one star**);

b) Subjects lost to follow up unlikely to introduce bias: number lost less than or equal to 20% or description of those lost suggested no different from those followed (**one star**);

c) Follow-up rate less than 80% and no description of those lost;

d) No statement.

<u>Case–Control Studies</u>

Selection (max. four stars):

1) Is the case definition adequate?

a) Yes, with independent validation (**one star**);

b) Yes, e.g., record linkage or based on self-report;

c) No description.

2) Representativeness of the cases:

a) Consecutive or obviously representative series of cases (**one star**);

b) Potential for selection biases or not stated.

3) Selection of controls:

a) Community controls (**one star**);

b) Hospital controls;

c) No description.

4) Definition of controls:

a) No history of disease (**one star**);

b) No description of source.

Comparability (max. two stars):

1) Comparability of cases and controls on the basis of the design or analysis, controlled for confounders:

a) The study controls for age (**one star**);
b) Study controls for other factors (tobacco/alcohol/localization of lesions) (**one star**).

Exposure (max. three stars):

1) Ascertainment of exposure:

a) Secure record (e.g., biopsy) (**one star**);

b) Structured interview, blind to case/control status (**one star**);

c) Interview not blinded to case/control status;

d) Written self-report or medical record only;

e) No description.

2) Same method of ascertainment for cases and controls:

a) Yes (**one star**);
b) No.

3) Non-response rate:

a) Same rate for both groups (**one star**);

b) Non-respondents described;

c) Rate different between cases and controls with no description.

Cross-Sectional Studies

Selection (max. four stars):

1. Representativeness of the sample:

   a. Truly representative of the average in the target population (all subjects or random sampling) (**one star**);

   b. Somewhat representative of the average in the target group (non-random sampling) (**one star**);

   c. Selected group of users/convenience sample;

   d. No description of the derivation of the included subjects.

2. Non-respondents:

   a. Proportion of target sample recruited attains pre-specified target or basic summary of non-respondent characteristics in sampling frame recorded (**one star**);

   b. Unsatisfactory recruitment rate, no summary data on non-respondents;

   c. No information provided.

3. Ascertainment of the exposure (risk factor):

   a. Validated measurement tool (**one star**);

   b. Non-validated measurement tool, but the tool is available or described (**one star**);

   c. No description.

Comparability (maximum two stars):

   1. Comparability of subjects in different outcome groups on the basis of design or analysis, confounding factors controlled;

   a. Data/results adjusted for relevant predictors/risk factors/confounders, e.g., age, sex, time since vaccination, etc.; **

   b. Data/results not adjusted for all relevant confounders/risk factors/information not provided.

Outcome (max. two stars):

1. Assessment of outcome:

   a. Independent blind assessment (**one star**);

   b. Record linkage (**one star**);

   c. Self-report;

   d. No description.

2. Statistical test:

   a. Statistical test used to analyse the data clearly described and appropriate, and measures of association presented including confidence intervals and probability level (p value) (**one star**);

   b. Statistical test not appropriate, not described or incomplete.

Table S1. NOS for quality rating of cohort studies.

Author, Year	A) SELECTION (max. 4 stars) (max. 4 stars)				B) COMPARABILITY (max. 2 stars)	C) OUTCOME (max. 3 star)			TOTAL STARS
	Representativeness of the exposed cohort (max. 1 star)	Selection of non-exposed cohort (max. 1 star)	Ascertainment of exposure (max. 1 star)	Demonstration that outcome was not present at start of study (max. 1 star)	Comparability of cohorts on the basis of the design or analysis (max. 2 stars); Study controls for age (= most important factor); Study controls for smoking (=additional important factor)	Assessment of outcome (max. 1 star); biopsy; record linkage; self-report only; no description	Was follow-up long enough for outcomes to occur (max. 1 star)?	Adequacy of follow-up of cohorts (max. 1 star)	
Kaur M et al. 2016			★		★★	★			4
Nanayakkara PG et al. 2016			★	★	★★	★			5
Remmerbach TW et al. 2017			★		★	★			3
Pandey P et al. 2018			★		★★	★	★		5
Velleuer E et al. 2020		★	★		★	★	★		5
Castillo P et al. 2022			★		★★	★	★	★	6
Bhatia N et al. 2014	★	★		★	★★	★	★		7
Francisco AL et al. 2014			★		★★	★			4
Scheer M et al. 2016			★		★	★	★		4
Simonato LE et al. 2019	★	★		★		★			4
Shukla A et al. 2018					★★	★			3
Vashisht N et al. 2014		★		★	★★	★	★	★	7
Lalla Y et al. 2016			★	★	★★	★			5
Grillone GA et al. 2017			★		★★	★	★	★	6
Malik A et al. 2017	★	★		★		★	★	★	6
Sharma D et al. 2021			★		★★	★	★	★	6
Awan KH et al. 2015				★	★★	★			4

Singh D et al. 2015				★	★★	★	★	★	6
Vijayakumar V et al. 2017			★		★★	★	★	★	6
Ali Channa S et al. 2019				★	★★	★		★	5
Algadi HH et al. 2020			★		★★	★	★	★	6
Jayasinghe RD et al. 2020			★	★	★★	★	★	★	7
Ma JM et al. 2014				★	★	★		★	4

Table S2. NOS for quality rating of cross-sectional studies.

Author, Year	A) SELECTION (max. 3 stars) (max. 3 stars)			B) COMPARABILITY (max. 2 stars)	C) OUTCOME (max. 2 stars)		TOTAL STARS
	Representativeness of the exposed subjects (OSCC/OPDM) (max. 1 star)	Non-respondents (max. 1 star)	Ascertainment of exposure (max. 1 star)	Comparability of exposed and non-exposed groups on the basis of the design or analysis (max. 2 stars):<br>a) Study controls for age (= most important factor)<br>b) Study controls for smoking (=additional important factor)	Assessment of outcome (biopsy/database) (max. 1 star)	Statistical test (max. 1 star)	
Jajodia E et al. 2016			★	★★	★	★	5
Neumann F et al. 2022		★	★	★	★		4
Kujan O et al. 2021			★	★	★	★	4
Idrees M et al. 2022			★	★★	★	★	5
Kaur J et al. 2015			★	★★	★	★	5
Messadi DV et al. 2014			★	★★	★	★	5
Ganga RS et al. 2017		★	★		★		3
Yamamoto N et al. 2017		★	★	★	★	★	5
Cânjău S et al. 2018		★	★	★★	★		5
Amirchaghmaghi M et al. 2018			★	★★	★	★	5

Chiang TE et al. 2019			★	★★	★	★	5
Morikaw T et al. 2020		★	★	★	★	★	5
Jain N et al. 2018		★	★	★★	★	★	5
Chaudhry A et al. 2016		★	★	★★	★	★	6
Knipfer C et al. 2014			★	★	★	★	4
Guze K et al. 2015			★	★★	★	★	5
Sircan-Kucuksayan A et al. 2020		★	★	★	★		4
Petruzzi M et al. 2014	★		★	★★	★	★	6
Nethan ST et al. 2018		★	★	★★	★	★	6
Iqbal W et al. 2023			★	★★	★	★	5
Trakroo A et al. 2014		★	★	★★	★	★	6
Gupta S et al. 2014		★	★		★	★	4

Table S3. NOS for quality rating of case–control studies.

Author, Year	A) SELECTION (max. 4 stars)				B) COMPARABILITY (max. 2 stars)	C) EXPOSURE (max. 3 stars)			TOTAL STARS
	Is the case definition adequate? (biopsy) (max. 1 star)	Representativeness of the cases (max. 1 star)	Selection of Controls (max. 1 star)	Definition of controls (max. 1 star)	Comparability of cases and controls on the basis of the design or analysis (max. 2 stars): a) Study controls for age (= most important factor) b) Study controls for smoking (=additional important factor)	Ascertainment of exposure (max. 1 star)	Same method of ascertainment for cases and controls (max. 1 star)	Non-response rate (max. 1 star)	
Sekine J et al. 2017	★	★			★	★		★	5
Goodson ML et al. 2016	★	★			★★	★		★	6
Alsarraf A et al. 2018	★	★		★	★★	★		★	7
Kokubun K et al. 2023					★★	★		★	4
Yuvaraj M et al. 2014	★	★		★	★	★		★	6
Simonato LE et al. 2017		★		★	★★	★	★	★	7
Murdoch C et al. 2014	★		★	★	★★	★		★	7

Brindha E. et al 2014	★			★	★	★	★	★	6
Krishna H et al. 2014	★			★	★★	★		★	6
Behl I et al. 2020	★			★	★★			★	5
Lejoy A et al. 2016	★			★	★★	★		★	6
Parakh MK et al. 2017	★	★		★	★★	★		★	7
Gupta M et al. 2019	★		★	★	★★	★		★	7

Table S4. NOS for quality rating of all studies

Type of study (N)	Low quality (score: 0-3)	Moderate quality (score: 4-6)	High quality (score: 7-9)
Cohort study (N=23)	2	18	3
Cross-sectional study (N=22)	1	21	-
Case series study (N=13)	-	8	5

G. Wells, B. Shea, D. O'Connell, J. Peterson, V. Welch, M. Losos, P. Tugwell

The Newcastle-Ottawa Scale (NOS) for Assessing the Quality of Nonrandomised Studies in Meta-Analyses [Internet]

Ottawa Hospital Research Institute (2011)

Available from:

http://www.ohri.ca/programs/clinical_epidemiology/oxford.asp

Google Scholar
